# Modulation of Zinc Homeostasis in *Acanthamoeba castellanii* as a Possible Antifungal Strategy against *Cryptococcus gattii*

**DOI:** 10.3389/fmicb.2017.01626

**Published:** 2017-08-24

**Authors:** Nicole S. Ribeiro, Francine M. dos Santos, Ane W. A. Garcia, Patrícia A. G. Ferrareze, Laura F. Fabres, Augusto Schrank, Livia Kmetzsch, Marilise B. Rott, Marilene H. Vainstein, Charley C. Staats

**Affiliations:** ^1^Programa de Pós-Graduação em Biologia Celular e Molecular, Centro de Biotecnologia, Universidade Federal do Rio Grande do Sul Porto Alegre, Brazil; ^2^Programa de Pós-Graduação em Microbiologia Agrícola e do Ambiente, Instituto de Ciências Básicas da Saúde, Universidade Federal do Rio Grande do Sul Porto Alegre, Brazil; ^3^Departamento de Biologia Molecular e Biotecnologia, Instituto de Biociências, Universidade Federal do Rio Grande do Sul Porto Alegre, Brazil; ^4^Departamento de Microbiologia, Imunologia e Parasitologia, Instituto de Ciências Básicas da Saúde, Universidade Federal do Rio Grande do Sul Porto Alegre, Brazil

**Keywords:** zinc, zinc transporters, *Cryptococcus gattii*, Acanthamoeba castellanii

## Abstract

*Cryptococcus gattii* is a basidiomycetous yeast that can be found in the environment and is one of the agents of cryptococcosis, a life-threatening disease. During its life cycle, cryptococcal cells take hold inside environmental predators such as amoebae. Despite their evolutionary distance, macrophages and amoebae share conserved similar steps of phagocytosis and microbial killing. To evaluate whether amoebae also share other antifungal strategies developed by macrophages, we investigated nutritional immunity against cryptococcal cells. We focused on zinc homeostasis modulation in *Acanthamoeba castellanii* infected with *C. gattii.* The intracellular proliferation rate (IPR) in amoebae was determined using *C. gattii* R265 and mutants for the *ZIP1* gene, which displays defects of growth in zinc-limiting conditions. We detected a reduced IPR in cells lacking the *ZIP1* gene compared to wild-type strains, suggesting that amoebae produce a low zinc environment to engulfed cells. Furthermore, flow cytometry analysis employing the zinc probe Zinpyr-1 confirmed the reduced concentration of zinc in cryptococcal-infected amoebae. qRT-PCR analysis of zinc transporter-coding genes suggests that zinc export by members of the ZnT family would be involved in the reduced intracellular zinc concentration. These results indicate that amoebae may use nutritional immunity to reduce fungal cell proliferation by reducing zinc availability for the pathogen.

## Introduction

*Cryptococcus gattii* and *Cryptococcus neoformans* are basidiomycetous yeasts that can be found in the environment and are the etiological agents of cryptococcosis, a life-threatening disease that is associated with nearly 200,000 annual deaths worldwide (Rajasingham et al., 2017). Yeast or spores are found in diverse ecological niches, especially in trees and soil, and are able to infect different hosts (May et al., 2016). Infection in mammalian hosts is initiated by the inhalation of airborne dehydrated yeast cells or spores that reach the lung and typically cause pneumonia or meningitis, which are driven by dissemination through the blood system (Harris et al., 2013). In the lung, alveolar macrophages initiate host defense by phagocytosis of the yeast cells. Despite the effectiveness of the host defense, *Cryptococcus* spp. developed virulence factors that allow them to inhibit and escape from the immune system. The best-characterized virulence factors in cryptococcal species include the production of a polysaccharide capsule, the synthesis of melanin and the secretion of enzymes that can destroy host cells. The production of such virulence factors thus allows cryptococcal survival in host cells and fluids (Coelho et al., 2014).

During its life cycle, *C. gattii* and its sibling species *C. neoformans* can also interact with other organisms in the environment, such as amoebae and nematodes (Springer et al., 2012). Free-living amoebae are protozoa that feed on both bacteria and fungi by phagocytosis (Guimaraes et al., 2016). *Acanthamoeba castellanii*, for instance, can phagocyte and digest *C. neoformans* in a similar mechanism that macrophages use when the latter enters a host system (Steenbergen et al., 2001). However, the yeast has also developed strategies to inhibit and escape from the amoeba antifungal repertoire. Cryptococcal cells are capable of killing amoebae, replicating inside the phagocytic vacuole and undergoing non-lytic exocytosis (Steenbergen et al., 2001). Both protozoan and mammalian phagocytes share common properties and strategies. During *C. neoformans* infection, both macrophage and amoeba cells engulf yeast cells within vacuoles, promoting the interaction of such phagosomes with other organelles and the secretion of lysosomal enzymes (Swanson and Hammer, 2000; Steenbergen et al., 2001; Bozzaro et al., 2008). The transcriptional responses of yeasts to protozoan or to macrophage ingestion are similar (Derengowski Lda et al., 2013). In fact, it was suggested that the antifungal mechanisms employed by free-living amoebae and macrophages are evolutionarily conserved, possibly due to a common ancestral between Metazoa and Amoebae (Siddiqui and Khan, 2012; Gaudet et al., 2016). Furthermore, it was shown that the interaction of pathogens such as *Cryptococcus* spp. and *Legionella pneumophila* with phagocytic cells in the environment have helped them to develop a repertoire of anti-phagocytic mechanisms to subvert the action of the mammalian host immune system (Bielska and May, 2016). Hence, it is currently assumed that *Cryptococcus* spp. developed its virulence toolkit under environmental selection by amoebae (Casadevall, 2012; Coelho et al., 2014; DeLeon-Rodriguez and Casadevall, 2016).

Several mechanisms are involved in the innate immune system of mammalian cells to avoid cryptococcal growth (Leopold Wager et al., 2016). Nutritional immunity is defined as a restriction of essential nutrients, including transition metals needed for pathogen development (Hood and Skaar, 2012). Zinc is the second most abundant transition metal in living organisms and is required in essential roles such as enzymes cofactors and structural constituents of proteins, in particular transcription factors (Hood and Skaar, 2012). We previously described that correct zinc metabolism regulation is important for *C. gattii* virulence in murine models of cryptococcosis (Schneider et al., 2012, 2015), reinforcing the importance of zinc uptake for proper cell metabolism. In addition, J774.A1 macrophages are also capable of decreasing zinc levels in response to *C. neoformans* infection (Dos Santos et al., 2017). This is similar to phenotypes observed for macrophages infected with different pathogens such as *Histoplasma capsulatum* (Haase, 2013) and *Candida albicans* (Lorenz et al., 2004; Crawford and Wilson, 2015). This suggests that zinc restriction should be considered a broad antifungal strategy (Crawford and Wilson, 2015).

Based on the fact that amoebae and macrophages share similar antifungal mechanisms and on the similarity of pathogenicity and behaviors between *C. neoformans* and *C. gattii* inside the host (Velagapudi et al., 2009), we hypothesized that amoeboid cells could also apply nutritional immunity as an antifungal strategy. We investigated the possible use by amoebae of a nutritional immunity mechanism, specifically zinc, as an antifungal strategy against *C. gattii*. We found that *A. castellanii* cells actively reduced zinc levels after exposure to *C. gattii*, possibly by mechanisms that include the activity of zinc exporters belonging to the ZnT family.

## Materials and Methods

### Strains and Growth Conditions

The *C. gattii* R265 (Kidd et al., 2004), the Δ*zip1* mutant, and the Δ*zip1*::*ZIP1* complemented mutant (Schneider et al., 2015) strains were used in this work. Yeast strains were routinely cultured in YPD medium (2% glucose, 2% peptone, and 1% yeast extract) and incubated in an orbital shaker (200 rpm) at 30°C for 18 h. *A. castellanii* strain *Neff* (ATCC 30010) was cultured in PYG (2% peptone; 0.2% yeast extract; 1.8% glucose, pH 6.5) supplemented with 100 U/ml penicillin and 100 μg/ml streptomycin, and incubated at 30°C.

#### Phagocytosis Index and Intracellular Proliferation Rate (IPR) Assays

To evaluate the phagocytosis index of *C. gattii* and fungal survival inside amoebae, protozoa cells were cultured in cell culture flasks, counted in a Neubauer chamber (1 × 10^5^ cells) and grown in 96-well plates containing PYG for 2 h to allow adhesion. *C. gattii* WT, Δ*zip1* and Δ*zip1*::*ZIP1* cells were inoculated in YPD medium for 18 h at 30°C. Cryptococcal cells were washed three times with phosphate buffered saline (PBS) and the cell density was evaluated in a Neubauer chamber. Yeast cells were inoculated at a ratio 10:1 with *A. castellanii* in PYG added or not of 10 μM ZnCl_2_. The incubation was allowed to proceed for 3 and 24 h. The wells were washed three times with warm PBS to remove non-phagocytosed *C. gattii* cells. Amoeba cells were lysed with 0.1% Triton X-100 (Sigma) to recover yeasts associated to amoeba cells. The intracellular proliferation rate (IPR) assay was performed using amoebae, as previously described for macrophages (Ma et al., 2009). Briefly, infection of amoebae was performed as described above. After 3 h of incubation, the amoeboid cells were washed with PBS. Amoebae of one set of wells were lysed 0.1% Triton X-100 and the number of associated yeasts was determined. Fresh YPG medium was added to another set of wells and interaction was allowed to occur for a further 24 h. Then, amoeba cells were washed with PBS and intracellular yeast cells determined. For both phagocytosis and IPR analysis, the lysates were diluted and plated on YPD-agar to analyze the number of colony forming units (CFUs). The IPR was defined as the ratio between CFUs recovered after 24 h incubation and the initial 3 h incubation.

Another set of experiments was performed to assess the effect of the presence of extracellular zinc on fungal replication and survival inside amoebae. The phagocytosis assay was allowed to proceed for 3 h under the same conditions as described above. Next, the interaction cells were washed with PBS and incubated with fresh medium containing 10 μM of zinc (ZnCl_2_) for a further 24 h. Amoeba cells were then lysed and the CFU analysis was performed in YPD solid agar to determine the IPR.

### *In Silico* Analysis

Sequences from *Mus musculus*, *A. castellanii*, and *Saccharomyces cerevisiae* belonging to the SLC39 and SLC30 transporter families were collected from Uniprot (UniProt Consortium, 2015) and AmoebaDB (Aurrecoechea et al., 2011). Such sequences were identified based on PFAM-conserved domain signatures ZIP (PF02535) and Cation_efflux (PF01545), respectively. We applied an OrthoMCL analysis (Altenhoff and Dessimoz, 2009) to identify orthologs of amoeba zinc transporters in yeast and mouse. Cellular localization was predicted using a Cell-PLoc server (Chou and Shen, 2008).

Phylogenetic analysis was conducted using protein sequences aligned by Clustal Omega (Sievers and Higgins, 2014) using the default options. The best fitting model of amino acid substitution was evaluated using ProtTest (Abascal et al., 2005) under the BIONJ JTT assumption. Bayesian inference was conducted using an LG+F+G+I model, while the MCMC sampling approach was used to calculate posterior probabilities. Four Markov chains were run 1,000,000 times. The chain was sampled every 100th generation, and burn-in values were determined from the likelihood values. The final tree diagram was generated using FigTree^1^.

#### RNA Extraction and Quantitative RT-PCR (qRT-PCR)

Total RNA was extracted from *A. castellanii* infected with *C. gattii* WT and Δ*zip1* after 3 and 24 h of interaction using Trizol^®^ reagent (Invitrogen) according to the manufacturer’s recommendations. RNA integrity was assessed by electrophoresis on a 1% agarose gel and RNA concentration was measured by spectrophotometry (NanoDrop 2000 spectrophotometer, Thermo Scientific). The samples were treated with RQ DNase (Promega) to purify RNA. Reverse transcription and cDNA synthesis were performed with ImProm-II Reverse transcriptase (Promega) using oligo-dT. The relative expression of genes identified as zinc transporters by the conserved domain (PF02535 and PF01545) of their coding products in *A. castellanii* were determined by qRT-PCR (StepOne Real-Time PCR System) with an initial step of 95°C for 10 min, followed by 50 cycles of 95°C for 15 s, 55°C for 15 s, and 60°C for 60 s. All experiments were performed in biological triplicate and each cDNA sample was also analyzed in technical triplicate for each primer pair. A melting curve analysis was performed at the end of the reaction to confirm the presence of a single PCR product. The results were processed according to the 2^-ΔCt^ method (Schmittgen and Livak, 2008) and relative transcript levels were normalized with actin transcript levels. The primers are listed in Supplementary Table S1.

#### Flow Cytometry Assay

To measure zinc levels in amoebae exposed to fungal cells, protozoa cells (1 × 10^5^) were grown in 12-well plates for 2 h at 30°C to allow adhesion to the surface. *C. gattii* WT and Δ*zip*1 cells were then added to the amoeba culture in a 10:1 ratio. The interaction was allowed to proceed for 24 h, after which the cells were washed three times with warm PBS. Attached cells were incubated with 20 μM of Zinpyr-1 fluorescent probe (Sigma) for 30 min at 30°C in the dark in PBS. Non-incorporated probe was removed by washing with PBS and the cells were collected from the well by cell scraper. Free zinc levels in amoeba cells were analyzed with a Guava easyCyte Flow Cytometer (Merck Millipore) by measuring the green fluorescence of 5,000 events.

### Fluorescence Microscopy

The lectin wheat germ agglutinin (WGA) was used to evaluate the chitin-like structures by fluorescence microscopy (Fonseca et al., 2009). Briefly, WT and Δ*zip1 C. gattii* cells were grown overnight in YPD broth, at 30°C and 200 rpm. Cells were recovered, washed with PBS and fixed with 4% paraformaldehyde in PBS for 30 min at room temperature. Fixed yeast cells were washed with PBS and blocked with 1% bovine serum albumine (BSA) in PBS, for 1 h, at 37°C. BSA solution was removed by washes with PBS and cells were then suspended in a 5 μg/mL solution of the Alexa Fluor 594 conjugate of WGA (Molecular Probes) and incubated for 30 min, at 37°C in the dark. After three consecutive washes with PBS, yeast cells were incubated with a calcofluor White (Invitrogen) solution at 5 μg/mL final concentration for 30 min, at 37°C in the dark. Cells were washed, suspended in 100 μL of PBS and placed onto glass slides containing glycerol plus *N*-propyl gallate. Images were analyzed and collected using an Olympus FluoView 1000 Confocal Laser Scanning Microscope (CME – UFRGS).

### Statistical Analysis

Data were expressed as mean ± standard deviation (SD) of replicates. All assays were performed in three experiment conditions, with technical triplicate repetitions. The Student’s *t*-test was employed to test for significance between values. *p*-values ≤ 0.05 were considered statistically significant.

## Results

### Zinc Uptake Is Important for *C. gattii* Survival in *A. castellanii*

**FIGURE 1:**
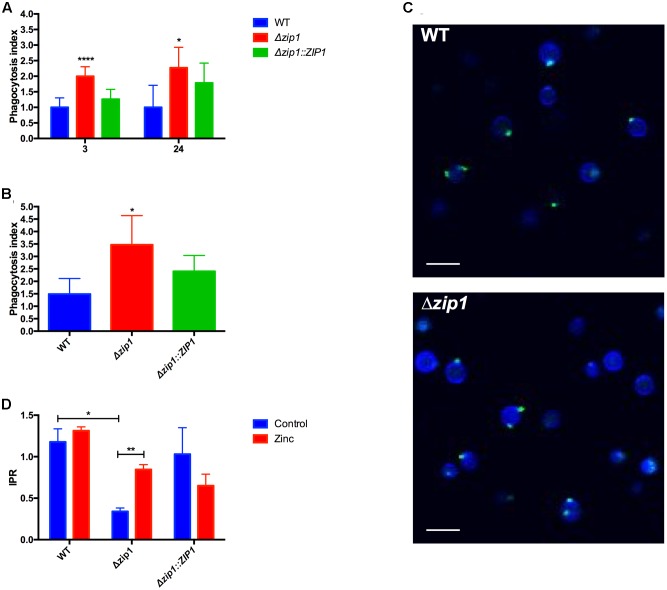
Absence of zinc transporter influences the outcome of *Cryptococcus gattii* from *Acanthamoeba castellanii.* **(A)** Cells of *A. castellanii* (1 × 10^5^) were incubated with WT, Δ*zip1* or Δ*zip1*::*ZIP1 C. gattii* strains (1 × 10^6^ cells) in PYG medium for 3 or 24 h in 96-well plates to allow phagocytosis. The wells were washed with PBS and *A. castellanii* cells were lysed. Yeast CFU count was assessed in YPD agar. The phagocytosis index was calculated using the total number of CFUs by the number of protozoan cells. Data are shown as mean ± SD. The asterisks denote statistically significant differences between the Δ*zip1* and the WT or Δ*zip1*::*ZIP1* conditions (^∗^*p* < 0.05; ^∗∗∗∗^*p* < 0.001 as revealed by Student’s *t*-test). **(B)** Influence of zinc on the phagocytosis was accessed by culturing cells of *A. castellanii* (1 × 10^5^) with WT, Δ*zip1* or Δ*zip1*::*ZIP1 C. gattii* strains (1 × 10^6^ cells) in PYG medium added of ZnCl_2_ (10 μM) for 24 h in 96-well plates. After washing, *A. castellanii* cells were lysed and yeast CFU count was assessed in YPD agar. Data are shown as mean ± SD. The asterisks denote statistically significant differences between the Δ*zip1* and the WT or Δ*zip1*::*ZIP1* conditions (^∗^*p* < 0.05; as revealed by Student’s *t*-test). **(C)** Fluorescence microscopy of WT and Δ*zip1* cells stained with Calcofluor White (Blue) and WGA (green) to access the surface structure of cryptococcal cells. Scale bars = 10 μm. **(D)** IPR assays were performed by incubating *A. castellanii* cells with WT or Δ*zip1 C. gattii* strains in PYG medium for 3 h in 96-well plates. After extensive washing to remove non-associated yeast cells, cells were incubated with medium added or not of ZnCl_2_ (10 μM). Interaction was allowed for a further 24 h, at 30°C. The *A. castellanii* cells were washed with PBS, lysed, and the yeast CFU count was performed in YPD agar. Data are shown as mean ± SD. The asterisks denote statistically significant differences between the conditions (^∗∗^*p* < 0.01 as revealed by Student’s *t*-test).

We recently provided evidence that *C. neoformans* cells experience zinc deprivation inside macrophages (Dos Santos et al., 2017). The main mechanism by which cryptococcal cells acquire zinc is through activity of the Zip1 protein (Schneider et al., 2015; Do et al., 2016). As null mutants of the *ZIP1* gene display severe growth impairment in zinc-limiting conditions, we hypothesized that *C. gattii Δzip1* strains could be used as biosensors to evaluate the modulation of zinc concentrations in *A. castellanii* cells exposed to *C. gattii*. The interaction between *A. castellanii* and *C. gattii* WT, Δ*zip1* mutant, and Δ*zip1*::*ZIP1* complemented strains showed that the mutant strain was more easily associated with amoebae compared to WT and complemented strain, independent of the extent of incubation (**Figure 1A**). We then sought to determine if the addition of zinc to the medium would alter the association of cryptococcal Δ*zip1* mutant strain to amoeba cells. We performed an interaction of amoebae and cryptococcal strains in the presence of zinc for 24 h, a period that would allow the cryptococcal Δ*zip1* mutant strain to properly acquire zinc. We could not detect a reversal of the higher number of Δ*zip1* mutant cells associated to amoeba compared to WT and complemented strains (**Figure 1B**). This suggest that zinc sufficiency is not responsible for alterations that led to this phenotype. In order to evaluate possible molecules associated with the higher phagocytosis sensitivity of cell lacking the *ZIP1* gene, we evaluated the distribution of chitin-like oligomers in the cryptococcal cells surface. These structures were show to be involved in the association of *C. neoformans* with murine phagocytes (Fonseca et al., 2013). Confocal fluorescent microscopy analysis revealed no differences in the staining pattern of chitin-like oligomers in WT or Δ*zip1* mutant strains (**Figure 1C**). This data suggest that other molecules present in cell surface could be affected in the absence of Zip1, which would increase the association of such cells with phagocytes.

We then analyzed whether the reduced capability of acquiring zinc from the extracellular environment could influence cryptococcal replication or survival inside phagocytic amoeba. IPR experiments were performed using WT, Δ*zip1* mutant, and Δ*zip1*::*ZIP1* complemented strains. We observed that the ability of *C. gattii* to survive and replicate inside amoebae was affected by the absence of *ZIP1 gene*, as Δ*zip1* mutant strain showed reduced recovery from amoebae when compared to WT and complemented strains (**Figure 1D**). This led us to hypothesize that engulfed cryptococcal cells experience a reduced zinc bioavailability. We first performed a growth curve analysis to rule out that the observed phenotype could be associated with a lower fitness of *C. gattii Δzip1* in PYG medium compared to the WT and complemented strains. No differences in growth were detected for such strains in PYG medium (data not shown). The IPR assay was repeated by including 10 μM of ZnCl_2_ in the interaction system, a concentration that did not alter the viability of the amoebae nor the cryptococcal cells according to MTT assays (data not shown). We were able to recover more CFUs from amoebae infected by *Δzip1* (**Figure 1D**). This suggests that addition of zinc to the media alters zinc bioavailability and promotes intracellular survival of *C. gattii* cells lacking the *ZIP1* gene.

### *C. gattii* Presence Alters Zinc Levels Inside Amoebae

**FIGURE 2:**
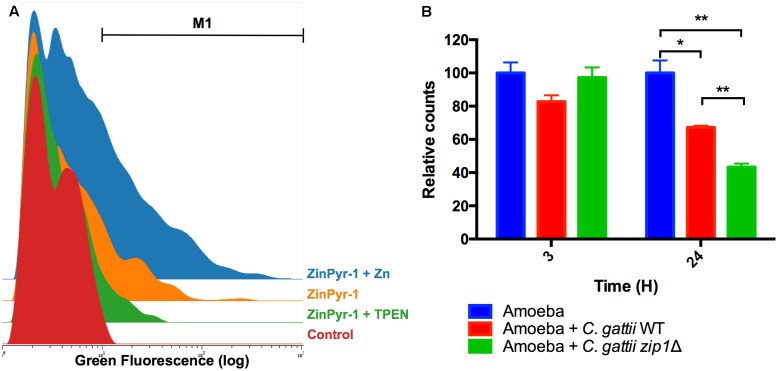
*Acanthamoeba castellanii* cells reduce intracellular zinc levels in the presence of *C. gattii*. **(A)** Cytometry histogram of ZinPyr-1 fluorescence *A. castellanii* cells cultured in PYG (Control), PYG plus 10 μM zinc chelator TPEN (TPEN) and PYG plus 50 μM ZnCl_2_. **(B)** *A. castellanii* (1 × 10^5^ cells) and *C. gattii* WT or *zip1*Δ (1 × 10^6^ cells) were incubated at 1:10 ratio in PYG medium for 3 and 24 h at 30°C. The wells were washed with PBS and then incubated with Zinpyr-1 cell-permeable fluorescent probe for 30 min. After, washes with PBS were performed and the cells were collected for flow cytometry analysis. Data are shown as the mean ± SD from three experimental replicates per condition. The asterisks denote statistically significant differences between the conditions, as evaluated by Student’s *t*-test (^∗^*p* < 0.05; ^∗∗^*p* < 0.01).

To investigate the reduction of zinc bioavailability to cryptococcal cells inside amoebae, we explored the labile zinc levels using the cell permeable fluorescent zinc probe ZinPyr-1. Despite the use of this probe to measure intracellular zinc levels in mammalian cells (Malavolta et al., 2006), little is known about zinc quantification in amoeboid cells. We therefore first validated our method by measuring ZinPyr-1 fluorescence in *A. castellanii* cells recovered from 2 h-cultures in PYG, PYG supplemented with 50 μM of ZnCl_2_ or PYG containing the zinc chelator TPEN (10 μM). Cytometry analysis revealed that ZinPyr-1 fluorescence was reduced when amoeba cells were exposed to TPEN and increased by the presence of ZnCl_2_, confirming the zinc-dependent fluorescence emission, as well the sensitivity of the assay (**Figure 2A**). We next evaluated whether intracellular zinc levels were reduced in amoeba cells exposed to *C. gattii* cells for distinct periods (3 and 24 h). Irrespective of the cryptococcal genotype used (WT or Δ*zip1*), a decrease in the number of cells in the gated region (M1) could be observed in amoeba cells incubated with *C. gattii* cells for 24 h, but not for 3 h (**Figure 2B**). We also noted that for Δ*zip1* mutant cells the reduction in zinc concentration was even more pronounced compared to that observed in amoeba cells exposed to WT cryptococcal cells, which could be due to higher Δ*zip1* mutant cells associated to amoebae. These results suggest that exposure of amoeba cells to *C. gattii* results in reduced intracellular zinc levels in the former.

### Identification and Function Assignment of ZIP and ZnT Proteins from *A. castellanii*

The SLC30 (ZnT) and SLC39 (ZIP) group of proteins are responsible for the maintenance of proper zinc levels inside cells (Stafford et al., 2013). We conducted a sequence comparison analysis in order to assign a possible function to the Zinc-transporting proteins coded by the amoeba genome. Analysis of the *A. castellanii*-predicted proteome for a PFAM-domain ZIP zinc transporter (PF02535) and Cation efflux family (PF01545) revealed the presence of 14 and 7 different proteins, respectively (**Table 1**). Analysis employing the OrthoMCL database revealed that both families of proteins (ZIP and ZnT) could be assigned to five different orthologous groups. In addition, the predicted subcellular localization analysis suggests that ZIP and ZnT proteins can occupy a range of different cell compartments (**Table 1**).

**Table 1:** Description of possible orthologs and predicted subcellular localization of amoeba zinc transporters.

Amoeba ID^1^	Localization^2^	OG^3^	Mouse sequences in OG^4^	Yeast sequences in OG^5^
ACA1_368320	Membrane	OG5_126707	Zip1, Zip3	Zrt1p, Zrt2p
ACA1_069540	Membrane			
ACA1_222780	Golgi			
ACA1_093920	Membrane			
ACA1_148440	Membrane	OG5_127397	Zip11	Zrt3p
ACA1_065010	Membrane			
ACA1_157200	Membrane			
ACA1_154170	Membrane			
ACA1_364600	Membrane			
ACA1_325560	Membrane	OG5_129531	Zip7	Yke4p
ACA1_271750	Membrane			
ACA1_100130	Membrane	OG5_138338	Zip6	ND
ACA1_385100	Membrane	OG5_239449	ND	ND
ACA1_358640	Membrane	ND^6^		
ACA1_107270	Peroxisome	OG5_126616	ND	ND
ACA1_260050	Secreted	OG5_126754	ZnT1, Znt2, ZnT4, ZnT10	Zrc1p, Cot1p,
ACA1_271600	Vacuole			
ACA1_038150	Membrane	OG5_128726	ND	Mmt1p, Mmt2p
ACA1_106270	Peroxisome			
ACA1_366570	Peroxisome	OG5_131446	ZnT9	ND
ACA1_191570	Membrane	OG5_135394	ZnT6	ND


*^1^Gene ID according to AmoebaDB. ^2^Predicted localization according to Cell-PLoc database. ^3^Ortholog group the sequence belongs to according to the OrthoMCL database. ^4^Mouse sequences belonging to the ortholog group according to the OrthoMCL database. ^5^Yeast sequences belonging to the ortholog group according to the OrthoMCL database. ^6^Not determined.*

**FIGURE 3:**
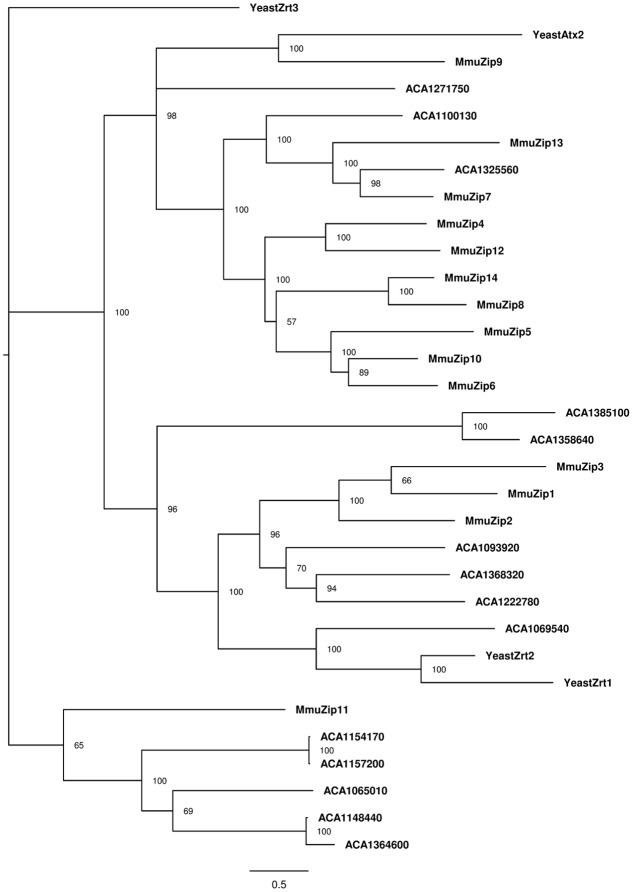
Phylogenetic reconstruction of ZIP zinc proteins from mouse, amoeba, and *Saccharomyces cerevisiae*. Sequences retrieved from Uniprot and AmoebaDB based on the presence of the Pfam domain ZIP (PF02535) were aligned with Clustal Omega, and the best evolutionary model was selected based on ProtTest. Bayesian analysis was conducted and the tree was drawn using FigTree. The percentage of replicate trees in which the associated taxa is shown next to the branches. The scale bar represents substitutions of amino acids per site.

In order to strengthen the OrthoMCL analysis, we verified the phylogenetically conserved level of these transporters by comparing them to ZIP and ZnT transporters from model organisms. We collected the zinc transporters from predicted proteomes of the yeast *Saccharomyces cerevisiae* (4 ZIPs and 5 ZnTs) and from the rodent *Mus musculus* (14 ZIPs and 10 ZnTs). The phylogenetic analysis of ZIP protein sequences showed that, with some exceptions, each amoeba protein has a close relationship with some proteins from the different analyzed organisms (**Figure 3**). We observed a division of such sequences into three major groups, which indicates a divergence in the evolution paths of these transporters. In the three clusters observed, it was possible to associate amoeba-specific zinc transporters with similar proteins from mouse or *S. cerevisiae*. These relations allow us to suggest that different sets of amoeba zinc transporters are closely related to transporters from different organisms. For instance, there are four *A. castellanii* proteins (ACA1_093920, ACA1_222780, ACA1_069540, and ACA1_368320) related to the main mammalian zinc importers (ZIP1 and ZIP2), as well with *S. cerevisiae* zinc importers (Zrt1p and Zrt2p). Five *A. castellanii* ZIP proteins (ACA1_065010, ACA1_157200, ACA1_154170, ACA1_364600, ACA1_148440) cluster with the mammalian ZIP11 transporter. The remaining *A. castellanii* ZIP proteins are related to several mammalian ZIP transporters (**Figure 3**).

**FIGURE 4:**
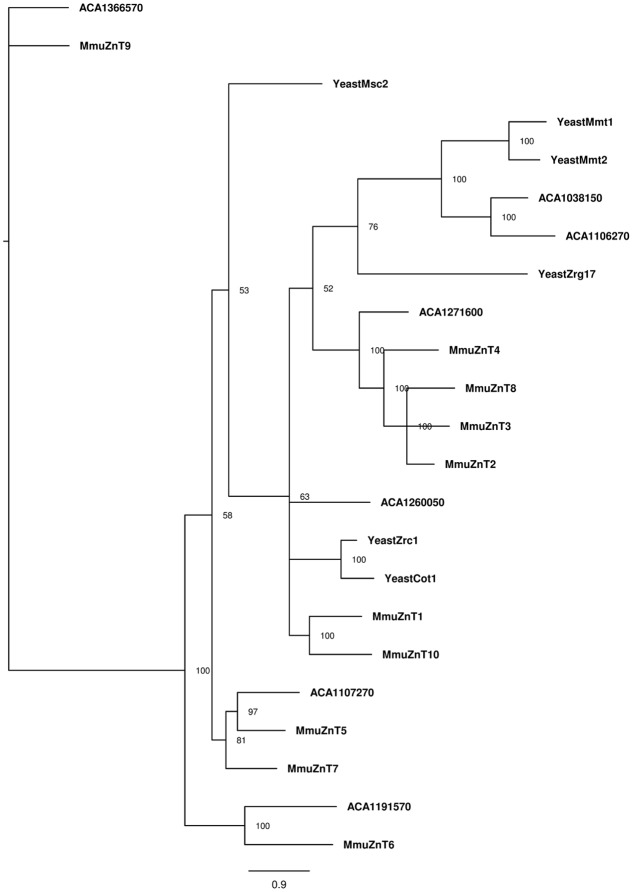
Phylogenetic reconstruction of ZnT zinc proteins from mouse, amoeba, and *S. cerevisiae*. Sequences retrieved from Uniprot and AmoebaDB based on the presence of Pfam domain ZnT (PF01545) were aligned with Clustal Omega, and the best evolutionary model was selected based on ProtTest. Bayesian analysis was conducted and the tree was drawn using FigTree. The percentage of replicate trees in which the associated taxa is shown next to the branches. The scale bar represents substitutions of amino acids per site.

A more complex pattern was observed for the phylogenetic analysis of ZnT protein sequences (**Figure 4**). The *A. castellanii* proteins ACA1_038150 and ACA1_106270 cluster with the *S. cerevisiae* mitochondrial iron transporters Mmt1 and Mmt2 (Li and Kaplan, 1997). In addition, ACA1_107270 from amoeba cluster with mammalian ZnT5 and ZnT7 Golgi-associated transporters (Kirschke and Huang, 2003; Thornton et al., 2011). The ACA1_191570 protein from amoeba is related to mammalian ZnT6, a protein that associates with ZnT5 and locates to the components of the early secretory pathway (Fukunaka et al., 2009). The transporter ACA1_271600 clusters with mammalian transporters ZnT2, ZnT3, ZnT4 and Znt8, proteins with multiple intracellular localizations (Lopez and Kelleher, 2009; Smidt and Rungby, 2012).

### Trancriptional Profiling of ZnT and ZIP Transporters Coding Genes in *A. castellanii* during Interaction with *C. gattii*

**FIGURE 5:**
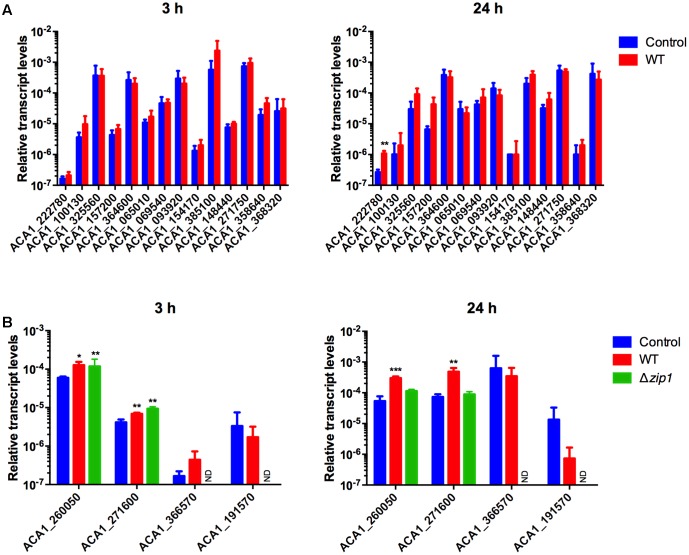
*Cryptococcus gattii* presence alters zinc transporter expression in *A. castellanii*. Amoebae (1 × 10^5^ cells) were incubated in PYG medium with *C. gattii* WT or *zip1*Δ (1 × 10^6^ cells) in 12-well plates for 3 and 24 h, at 30°C. The cells were washed with PBS and RNA from amoebae was isolated and extraction was performed, followed by cDNA synthesis. The measured quantity of zinc transporter mRNA from ZIP coding genes **(A)** or ZnT coding genes **(B)** in each sample was normalized using the threshold cycle values obtained for the *Actin* gene. Data are shown as the mean ± standard deviation from three experimental replicates of three biological replicates. Statistical analysis by *t*-student comparing control and fungal presence groups for each transporter (^∗^*p* < 0.05, ^∗∗^*p* < 0.01, ^∗∗∗^*p* < 0.001). ND, non-determined.

We next analyzed the transcriptional profiling of 18 genes (14 ZIPs and 4 ZnTs) using qRT-PCR. We performed such experiments in order to evaluate whether alterations in zinc concentrations inside amoebae in response to cryptococcal presence was associated with the activity of zinc transporters. cDNA was synthesized from RNA samples collected from amoeba cells co-incubated or not with *C. gattii* WT for 3 and 24 h. Irrespective of the condition analyzed, we detected transcripts from ZIP transporter-coding genes. However, only ACA1_222780 was found to be differentially expressed when compared amoebae exposed or not to cryptococcal cells. This increase in expression could only be detected after 24 h of co-incubation (**Figure 5A**).

When considering the genes coding for ZnT family zinc transporters, only four genes were investigated due to phylogenetic proximity to known mammalian genes possibly involved in nutritional immunity. Despite the detection of transcripts for all analyzed genes, two of them were observed to be differentially expressed. We found increased levels of transcripts from genes ACA1_260050 and ACA1_271600 in amoebae co-incubated with cryptococcal cells compared to control conditions for both times analyzed (**Figure 5B**). As the labile pool of zinc was even lower in amoeba cells exposed to the Δ*zip1* mutant strains, we also evaluated the transcript levels of zinc exporters in such cells. For a 3 h-incubation period of amoebae and Δ*zip1* cells, we could detect increased transcript levels of the genes ACA1_260050 and ACA1_271600 compared to control conditions. However, after 24 h of co-incubation, the transcript levels of such genes return to control levels (**Figure 5B**). Collectively, these results suggest that amoeba cells are exporting zinc to extracellular spaces in order to hamper cryptococcal development.

## Discussion

Amoebae share diverse features with mammalian macrophages, including the capability of undergoing phagocytosis, producing phagosomes, and killing microbes through a combination of lytic enzymes and low pH (DeLeon-Rodriguez and Casadevall, 2016; Leopold Wager et al., 2016). Among the mechanisms by which amoebae are capable of reducing microbial growth is the modulation of metal homeostasis. A strong correlation is described for the capability of bacterial cells to metabolize different metals due to the presence of copper/zinc efflux as well as iron/manganese uptake proteins, and their resistance to amoeba bactericidal activity (Hao et al., 2016). In fact, the same phenotype was observed for bacterial capability to grow inside human macrophages (White et al., 2009; Botella et al., 2011; Achard et al., 2012). This reinforces the hypothesis that intoxication of bacterial cells inside phagosomal compartments with copper or zinc represents an evolutionary bactericidal activity of phagocytes (German et al., 2013).

When considering the interaction of amoeba species with fungal species, little is known about the molecular mechanisms by which amoebae impair fungal growth. Transcriptional profiling of *C. neoformans* cells recovered from *A. castellanii* and murine macrophages revealed a common set of genes that were upregulated. Among these genes were those involved in nutrient uptake (Derengowski Lda et al., 2013). It is therefore reasonable to assume that cryptococcal cells inside phagocytic cells experience nutrient deprivation. This is a well-described innate immune response, termed nutritional immunity, that is characterized by the activity of a range of proteins acting to reduce the bioavailability of nutrients necessary for pathogen growth (Kehl-Fie and Skaar, 2010; Hood and Skaar, 2012). Zinc restriction has already been characterized for macrophages infected with the fungal pathogens *H. capsulatum* and *C. neoformans*. The mechanism by which zinc levels were reduced in macrophages was shown to be dependent on the activity of zinc transporters of the ZnT family (Winters et al., 2010; Subramanian Vignesh et al., 2013a; Dos Santos et al., 2017). At least four lines of evidence allowed us to conclude that amoebae expose cryptococcal cells to a nutritional immunity-like mechanism: (i) assays employing the zinc fluorescent probe ZinPyr-1 revealed that labile zinc levels were reduced in *A. castellanii* cells exposed to *C. gattii*; (ii) transcription levels of some *A. castellanii* ZnT-coding genes were highly modulated by the presence of cryptococcal cells; (iii) *C. gattii* cells with reduced growth in zinc-limiting conditions experienced impaired proliferation inside amoeba cells; and (iv) addition of extracellular zinc enhanced intracellular survival of *C. gattii* cells lacking the major zinc transporter. Therefore, it is feasible to assume that nutritional immunity could be another conserved antifungal mechanism shared by phagocytic cells.

One remarkable observation associated to the absence of *ZIP1* gene in *C. gattii* is the higher association to amoeboid cells. In fact, this phenotype was already observed during interaction with the J774.A1 mammalian macrophage-like cells (Schneider et al., 2015). Here, we provided extra evidence that the absence of Zip1 does not drive substantial alterations in cryptococcal cell wall, whose components are supposed to be involved in the recognition by phagocytic cells (Garcia-Rodas and Zaragoza, 2012). For instance, the distribution of chito-oligomers in cell surface is directly involved in the yeast sensitivity to phagocytosis, at least in mammalian cells (Ost et al., 2017). We cannot observe significant differences in WGA-stained chito-oligomers distribution in cell walls of WT and *ZIP1* null mutants. As Zip1 from the sibling species *C. neoformans* localizes in plasma membrane (Do et al., 2016), the participation of this protein as a ligand for a putative receptor present in phagocytic cells should be minor. So, we propose that the higher association of *zip1*Δ cells to amoebae is associated to structural changes in the cell wall that could not be reversed by a short-term zinc exposure during interaction. It is important to note that defects of zinc homeostasis regulation in cryptococcal cells lacking the master zinc regulator Zap1 also leads to higher association to RAW 264.7 macrophage-like cells (Schneider et al., 2012), reinforcing the association of zinc regulation and association with phagocytic cells. However, the molecular mechanisms that lead to this higher association remains to be elucidated.

Macrophages modulate the expression of zinc transporters of the ZnT family and metallothioneins in order to reduce zinc bioavailability for *H. capsulatum* (Subramanian Vignesh et al., 2013a) and *C. gattii* (Dos Santos et al., 2017). Among such transporters, ZnT4 and ZnT7 are assumed to play a direct role in zinc compartmentalization, as the transcript levels of such genes were regulated in macrophages infected with fungal pathogens (Subramanian Vignesh et al., 2013a; Dos Santos et al., 2017). These proteins are associated with the Golgi apparatus (Subramanian Vignesh et al., 2013b; Subramanian Vignesh and Deepe, 2016). Due to the activity of such proteins, macrophage cells infected with *H. capsulatum* mobilize zinc into the Golgi (Subramanian Vignesh et al., 2013a). Our *in silico* analysis to assign a possible function to zinc transporters coded by the amoeba genome allow us to speculate that the products of genes ACA1_260050 and ACA1_271600 could function as orthologs of mammalian ZnT4, driving the mobilization of zinc to the Golgi. In line with this assumption, the transcript levels of ACA1_260050 and ACA1_271600 increase in amoeba cells exposed to *C. gattii*. Zinpyr-1 is a zinc probe mainly used to detect zinc associated to Golgi apparatus in mammalian cells (Lu et al., 2016). Our assays using this probe to detect zinc in amoeba cells revealed a very diffuse signal instead of compartmentalized fluorescence (data not shown), suggesting that Zinpyr-1 use in *A. castellanii* cells could be used to quantify the labile pool of zinc present in all cellular compartments. As a reduced free pool of zinc was observed in amoeba cells exposed to *C. gattii*, it is possible that such gene products may also localize to the plasma membrane and export zinc to the extracellular space. Alternatively, the participation of zinc-chelating proteins cannot be ruled out.

The capability of fungal pathogens to proliferate inside amoeba cells has been associated with the development and/or acquisition of phenotypes that correlate with the virulence potential of such pathogens in mammalian hosts (Casadevall, 2012; Guimaraes et al., 2016). Zinc is essential to all life forms and its acquisition is mainly mediated by the activity of proteins from the ZIP family of zinc transporters (Jeong and Eide, 2013). We have described that zinc acquisition in *C. gattii* is performed mainly by Zip1. It is noteworthy that *C. gattii* cells lacking Zip1 displayed reduced proliferation inside macrophages (Schneider et al., 2015). The same phenotype was observed in the sibling species *C. neoformans* (Do et al., 2016). This appears to be a conserved characteristic in fungal species, as a clear association was observed in zinc uptake regulation and virulence in some *Candida* species and *Aspergillus fumigatus* (Ballou and Wilson, 2016). The results presented here suggest that zinc acquisition is important for cryptococcal proliferation inside amoebae. This implies that the development of efficient zinc acquisition strategies by fungal pathogens during their evolution has been important for successful infection inside a wide range of hosts. Cryptococcal cells are capable to reside inside phagocytes and to produce molecules that modulate the activity of such cells in order to aid cryptococcal survival and growth, exemplified by the permeabilization of phagosome membrane that would provide access to nutrients from cytoplasm (Steenbergen et al., 2001; Tucker and Casadevall, 2002). This is possibly also true for other fungal pathogens that can reside inside amoeba cells (Casadevall, 2012; Guimaraes et al., 2016). We noted that cryptococcal cells lacking the *ZIP1* gene elicited a more pronounced reduction of zinc bioavailability in amoeba cells. We speculate that the reduced fitness of *zip1*Δ mutant cells associated to amoebae could be linked to this phenotype. As cryptococcal cells lacking the *ZIP1* gene are more sensitive to amoeba antifungal activity, the modulatory properties of cryptococcal cells would not be active at the same extent in mutant cells compared to WT cells. So, we hypothesize that either the higher number of *zip1*Δ cells associated to amoebae, together with the reduced fitness of such strain to modulate the antifungal activity of host cells led to an even higher modulation of zinc bioavailability to the pathogen.

## Conclusion

The data collected here allow us to propose that zinc bioavailability modulation, driven by mechanisms that include the activity of zinc transporters of the ZnT family, is a conserved antifungal mechanism. However, functional assignment of such proteins is a further necessary step to support this hypothesis.

## Author Contributions

NR, FdS, AG, PF, LF, AS, LK, MV, MR, and CS prepared the experimental design. NR, FdS, and LF conducted the CFU analysis. NR and PF performed the *in silico* analysis. NR and AG performed the qRT-PCR analysis. NR and FdS performed the flow cytometry analysis. NR, LK, AS, MV, MR, and CS discussed the results and wrote and approved the final manuscript.

## Conflict of Interest Statement

The authors declare that the research was conducted in the absence of any commercial or financial relationships that could be construed as a potential conflict of interest.

## Footnotes

1 http://tree.bio.ed.ac.uk/software/figtree/
