# Supplementary material for: Modulation of Zinc Homeostasis in Acanthamoeba castellanii as a Possible Antifungal Strategy against Cryptococcus gattii
Source: Front Microbiol. 2017 Aug 24;8:1626. doi: 10.3389/fmicb.2017.01626 (PMC5573748; doi:10.3389/fmicb.2017.01626)
Supplement: Supplementary file 1 [file Table_1.DOCX]

**Supplementary material**

**Table S1**: List of primers used in this work.

| Primer ZIPs | Sequence (5’ -  3’) | NCBI Gene ID |  |
| --- | --- | --- | --- |
| **ACTIN-F** | AGGTCATCACCATCGGTAACG | 14922056 |  |
| **ACTIN-R** | TCGCACTTCATGATCGAGTTG |  |  |
| **ACA1_325560-F** | AGGAGAAGAAGAAGAGGGAGAG | 14922904 |  |
| **ACA1_325560-R** | ATGGCAAAGTCACCGATCTC |  |  |
| **ACA1_100130-F** | ACCTCGTGGTGATGCTTATG | 14913735 |  |
| **ACA1_100130-R** | TGTGGTGGAGACAAAGTGAG |  |  |
| **ACA1_157200-F** | CCTCTGTTTCTTCTCCGGC | 14922918 |  |
| **ACA1_157200-R** | TTGTGGACGCAAATAGCGA |  |  |
| **ACA1_222780-F** | TTGGGCATTCTACTGGGTTC |  |  |
| **ACA1_222780-R** | AGGAGGAACTTGATGTACTTGTC | 14916731 |  |
| **ACA1_368320-F** | GACGGAAGAGGTATCCGCTG |  |  |
| **ACA1_368320-R** | GTTCCAGAAGAGGACGCCAA | 14918922 |  |
| **ACA1_093920-F** | TTTCCTCCATCTCCAGCATTC |  |  |
| **ACA1_093920-R** | CACCACCGAGACAGTTCATC | 14913245 |  |
| **ACA1_069540-F** | TTCGGCCTTCTGTTCCATAAC |  |  |
| **ACA1_069540-R** | CTCGAAGAACTGGTGGAAGAT | 14924343 |  |
| **ACA1_154170-F** | CGTCGAGATCCTCACCAAATC |  |  |
| **ACA1_154170-R** | GGAGAAGAAACAGAGGGAAGTG | 14919351 |  |
| **ACA1_271750-F** | CTCGTGCCCAAGTTCATCTATC |  |  |
| **ACA1_271750-R** | GTGGCGATGGCGAAGAG | 14915206 |  |
| **ACA1_385100-F** | CCGCTCCTGACCAAACTG |  |  |
| **ACA1_385100-R** | GTCCTCCATTTCCTTCTCTTCTC | 14922666 |  |
| **ACA1_065010-F** | GGTGCCGATTGCCTACA |  |  |
| **ACA1_065010-R** | GAGTTCGTTGAGCGAGATGA | 14918388 |  |
| **ACA1_148440-F** | CCTACACCTGGCTCTTCTTTG |  |  |
| **ACA1_148440-R** | AAAGTGCGGTTCGGGTATG | 14923782 |  |
| **ACA1_364600-F** | CATGTGCATCCACAACTTGC |  |  |
| **ACA1_364600-R** | AACCCACAACGAACTCCTTC | 14914461 |  |
| **ACA1_358640-F** | AGAAAGCGTCTTCTGGAGTG |  |  |
| **ACA1_358640-R** | CTCTTCCAACTCCTGCTCTATC | 14914121 |  |

| Primer Znts | Sequence (5’ -  3’) | NCBI Gene ID |
| --- | --- | --- |
| **ACA1_260050-F** | ACGAAGAGAGCCACTACCA | 14912155 |
| **ACA1_260050-R** | CATCGAGATCCCACACCTTTAC |  |
| **ACA1_271600-F** | CGCTTGTGGCTATGGGTATC | 14915201 |
| **ACA1_271600-R** | GTACTTGATGTCCGCGATGAG |  |
| **ACA1_191570-F** | GTTGATGGCGTTGAGCTTCC | 14915050 |
| **ACA1_191570-R** | CCGTAGGTGTAGCTAGGGGT |  |
| **ACA1_366570-F** | GATTCTCGTCTCTCACCATACAC | 14914485 |
| **ACA1_366570-R** | GGGTAGTTCTTGTCGATGTACC |  |
